# Relationship between oxidative stress and haematological indices in patients with diabetes in the Ghanaian population

**DOI:** 10.1186/s40842-015-0008-2

**Published:** 2015-08-20

**Authors:** RH Asmah, G Yeboah, H Asare-Anane, S Antwi-Baffour, TN Archampong, CA Brown, G Amegatcher, DN Adjei, B Dzudzor, J Akpalu, PF Ayeh-Kumi

**Affiliations:** 1 Department of Medical Laboratory Sciences, School of Biomedical and Allied Health Sciences, University of Ghana, Korle-bu, Accra, Ghana; 2 Department of Chemical Pathology, School of Biomedical and Allied Health Sciences, University of Ghana, Korle-bu, Accra, Ghana; 3 Department of Medicine, School of Medicine and Dentistry, Korle-bu, Accra, Ghana; 4 Department of Medical Biochemistry, School of Biomedical and Allied Health Sciences, University of Ghana, Korle-bu, Accra, Ghana

**Keywords:** Reactive oxygen species, Superoxide dismutase, Diabetes Mellitus, Haematological parameters

## Abstract

**Background:**

Persistent hyperglycaemia is a hallmark of Diabetes Mellitus (DM). It causes increased production of free radicals, especially reactive oxygen species (ROS), — resulting in oxidative stress. Reactive Oxygen Species have been implicated in the development of haematological complications in patients with diabetes. Superoxide Dismutase (SOD) is one of the most effective antioxidant enzyme defense systems against free radicals.

**Methods:**

From February through May 2014, we assessed the relationship between oxidative stress and haematological profiles among individuals with and without diabetes. A cross sectional study of 66 case patients and 44 age-matched controls were recruited from the National Diabetes Management and Research Centre (NDMRC), Korle-Bu Teaching Hospital, Accra, Ghana. Blood samples were obtained from study participants with consent. We determined the haematological profiles of study participants and measured their oxidative stress levels using a standardized kit for SOD activity.

**Results:**

Higher white blood cell (WBC) counts were seen in the diabetes cohort (*p*-value = 0.023). The SOD activity tended to be lower in diabetes patients (p–value = 0.144 however) while higher neutrophil levels seemed to correlate with SOD activity (R = 0.249; R^2^ = 6.2 %; *p*-value = 0.049). There did not appear to be a correlation between fasting blood glucose (FBG) and SOD activity (R = −0.044; *p*-value = 0.727).

**Conclusion:**

The study reports similar oxidative stress levels, as measured by SOD activity, in diabetic and non-diabetic adults. The SOD activity did not appear to correlate with FBG and several other haematological parameters. Further study would be required to investigate the relationship between these haematological indices and diabetic micro- and macro-vascular complications in our population.

## Background

Diabetes mellitus (DM) is major cause of adult morbidity and death in Ghana — with an estimated prevalence of 3.3 % that represents a diabetic population of 354,020 [1]. The disease is characterized by a deficiency in insulin resulting in disturbances in carbohydrate, lipid and protein metabolism, which manifest as chronic hyperglycaemia. Persistent hyperglycaemia in DM causes an increased production of free radicals especially Reactive Oxygen Species (ROS). Destruction mediated by such free radicals results in protein denaturation, DNA strand breaks and alterations of cell membrane fluidity. Antioxidant enzyme defense systems against free radicals include superoxide dismutase (SOD) and catalase [2, 3]. Superoxide Dismutase is one of the most effective intracellular enzymatic antioxidants that catalyzes the conversion of superoxide (O_2_^−^) to oxygen (O_2_) and to the less reactive species, hydrogen peroxide (H_2_O_2_) [4, 5]. Reports indicate that antioxidants are either reduced or not sufficient to eliminate ROS in diabetic patients leading to oxidative stress [6]. Oxidative stress results from an imbalance between free radicals and the body’s antioxidant defense systems; resulting in red blood cell dysfunction, platelet destruction and tissue injury. In patients with diabetes, such abnormalities may affect the functions of blood cells as well as the coagulation system leading to complications such as anaemia, infection, and hypercoagulability [7]. Yet, these haematological changes also opens up the possibility that circulating blood cells, particularly leukocytes including lymphocytes and neutrophils could serve as biomarkers for oxidative stress, and as an index of diagnostic significance in routine clinical evaluation of the state of diabetes [8]. We conducted this study with a two-fold primary objective. First, to determine whether patients with diabetes have higher oxidative stress, and second to evaluate the association between oxidative stress and certain changes in hematological indices.

## Methods

### Study site

This study was carried out at the National Diabetes Management and Research Centre (NDMRC), Accra, Ghana. It serves as the Diabetes Clinic of the Korle-Bu Teaching Hospital (KBTH). The KBTH is a 2000-bed tertiary teaching hospital with about 200 admissions per day. The hospital covers all medical specialties and provides referral healthcare services to an estimated population of 24 million Ghanaians. The NDMRC is designed to manage diabetic conditions of patients in Ghana. The Centre also supports and enhances the national effort in diabetes, its complications, and related endocrine and metabolic diseases through interdisciplinary research.

### Study population

The study population comprised Diabetes Mellitus patients aged 30 to 80 years who attended the NDMRC and consented to participate in the study. A volunteer group without Diabetes Mellitus and of the same age-brackets were enrolled as controls.

### Ethical approval

Ethical approval was obtained from the Ethics and Protocol Review Committee of the School of Allied Health Sciences, University of Ghana prior to the enrolment of participants.

### Study design

This was an analytical cross-sectional study with age-matched comparison controls. The study was conducted from February through May 2014. Two sampling approaches were used. First, an active case-finding network was organized with visits to the NDMRC at Korle-Bu. Physician-identified patients with known type-1 or type-2 diabetes attending the center for review and management of various diabetic conditions were referred to the research team for screening and inclusion in the study after informed consent was obtained. Diabetic patients enrolled into the study were categorized as cases. Prior to enrollment, the nature, purpose and potential risks of the study were explained to all subjects. Patients’ case history was ascertained through physician-assisted review of medical folders. Cases were recruited based on the following screening criteria: 30–80 years of age, pre-prandial capillary plasma glucose levels of 3.9 – 7.2 mmol/l and mean plasma glucose of 8.6 mmol/l correlating with achievement of target glycated haemoglobin (HbA1c) of <7 % as per the [9] criteria. Overall 66 patients were enrolled to participate in the study. Forty-four other subjects without diabetes were prospectively recruited as controls within the same study period. The control group were aged-matched volunteers accompanying cases to the diabetic center. The same biochemical criteria were examined in the controls subjects. Controls had fasting plasma glucose <5.6 mmol/L or two-hour glucose during OGTT < 7.8 mmol/L. Second, venous blood samples were collected from diabetics and non-diabetic controls and analyzed for various haematological changes.

### Exclusion criteria

Pregnant and lactating women were excluded from the study. Patients with medical conditions such as infections, cerebrovascular diseases, ischaemic heart disease and malignancies excluded from the study. Other exclusion criteria were smoking and alcoholism.

### Study conduct

#### Haematological indices and fasting glucose analysis

After an overnight fast, 5 ml of venous blood was collected from each participant. Three milliliters of which was put into an EDTA tube for haematological indices and SOD activity analysis and 2 ml was put into a fluoride tube for fasting plasma glucose (FPG) analysis using an automated analyzer (A25 Biosytems, Canada). The haematological indices were determined by performing a full blood count (FBC) using an automated haematology analyzer (Sysmex KX-21 N, Germany). The following haematological indices were assessed: Haemoglobin, Haematocrit, Mean cell volume, white cell count (including lymphocyte and neutrophil count); platelet count. The rest of the blood sample was then centrifuged at 4000 rpm for 10 min to separate plasma from the red blood cells. The stored red blood cells were used for further work involving SOD activity determination.

#### Measurement of sod activity as an index of oxidative stress status

Briefly, the SOD activity levels were determined by using SOD Assay kit (Cayman Chemicals, Michigan, USA). Whole blood obtained by venipuncture from the participants was centrifuged at 4000 rpm for 10mins, and plasma carefully separated. Two hundred and fifty (250) microliters of erythrocytes obtained from the packed cells was lysed with thousand (1,000) microliters of ice-cold deionized distilled water and centrifuged at 6,000 rpm for 20 min. Thousand microliters (1000 μl) of the supernatant (erythrocyte lysate) was collected for assay and stored on ice. The supernatant fluid was then diluted by a factor of 100 with sample buffer, and 10 μl of the diluted solution used to assay Cu/ZnSOD (Copper/Zinc Superoxide dismutase) activity as described by the kit manufacturer.

### Data analysis

Data obtained was put into a database and analyzed statistically using Statistical Package for Social Sciences (SPSS) 20.0 software for windows. Summary statistics was presented using the descriptive statistics of mean and standard deviation for numerical variables and proportions for categorical variables. Independent t-test or analysis of variance (ANOVA) and Pearson’s correlation analysis were used to determine the mean differences and the correlation between oxidative stress and haematological parameters respectively. A *p*-value less than 0.05 was interpreted as significant.

## Results

### Demographic and clinical data

One hundred and ten (110) participants were recruited to the study — consisting of 66 subjects with diabetes (cases) and 44 subjects without diabetes (controls). For both cases and controls, over 80 % were made up of females. The mean ages for cases and controls were 55.85 ± 8.82 years and 50.32 ± 12.44 years respectively (Table 1). Fasting plasma glucose concentration was higher among the cases compared with the controls (p = 0.000). In this study, cases were mostly on stable doses of Glibenclamide, Metformin and Insulin in various combinations. Among patients with diabetes, 24.2 % (n = 16) were on a combination of Glibenclamide and Metformin while 22.7 % (n = 15) used both metformin and Insulin. About 40 % (n = 26) of cases were on metformin alone for the management of diabetes. Overall, 9 diabetic patients (n = 13.6) used other diabetic drugs aside metformin.

**Table 1:** Summary of clinical parameters in diabetic subjects in comparison to controls

Parameters	Number of cases	Number of controls
Number	66	44
Female/male	55/11	38/6
Mean Age (years) ± SD	55.85 ± 8.82	50.32 ± 12.44
FBG (mmol/L) ± SD	8.34 ± 3.47	4.75 ± 0.55
Duration of Diabetes (years) ± SD	10.67 ± 7.53	**-**
Anti-hypertensive drugs	39 (59.0 %)	8 (18.1 %)
Patients on Metformin only	26 (39.4 %)	**-**
Patients on Metformin plus other drugs	57 (83.6 %)	**-**
Patients Metformin plus Glibenclamide	16 (24.2 %)	**-**
Patients Metformin plus Insulin	15 (22.7 %)	**-**
Patients on other drugs aside metformin	9 (13.6)	**-**

SD, standard deviation

### Oxidative stress

Table 2 shows the SOD activity values analyzed on blood samples obtained from the participants. Superoxide dismutase activity among case patients (285.44 ± 114.36 U/ml) did not significantly differ (*p*-value 0.144) from those observed within the control group (319.79 ± 126.12 U/ml). When SOD activity was compared within case patients, we observed homogeneity in SOD activity values between patients using metformin only, those on diabetic medications aside metformin, and those taking metformin in combinations with other drugs (degree of freedom, 2; F-value, 0.261; *p*-value, 0.771).

**Table 2:** Superoxide dismutase activity in diabetic subjects in comparison to controls

Parameters	Cases ± SD	Controls ± SD	*p* value
Total SOD Activity (U/ml)	285.44 ± 114.36	319.79 ± 126.12	0.144
SOD Activity (U/ml) of cases on Metformin only^*a*^	274.81 ± 122.73	-	a*b*c =0.771
SOD Activity (U/ml) of cases on Metformin and other drugs^*b*^	281.75 ± 111.91	-	*Post hoc:*
SOD Activity (U/ml) of cases on other drugs aside Metformin^*c*^	307.98 ± 133.40		a*b =0.824 ;
			a*c =0.499;
			b*c =0.5265

FBG- Fasting blood glucose; SOD-Superoxide dismutase; SD, standard deviation

### Haematological parameters

Table 3 compares various haematological indices between diabetic patients and the control cohort. There was a significantly higher (p = 0.023) total white blood cell count in the diabetes cohort compared to control subjects. There did not appear to be differences in lymphocyte count, neutrophil count, red cell count, platelet count, haemoglobin and haematocrit levels between diabetic cases and the control group.

**Table 3:** Full blood count of study participants

Parameters	Cases ± SD	Controls ± SD	*p* value
WBC (x10^3^/μL)	5.87 ± 1.76	5.25 ± 1.05	0.023*
RBC (x10^6^/μL)	4.48 ± 0.60	4.57 ± 0.54	0.426
HGB (g/dL)	12.29 ± 1.50	12.60 ± 1.30	0.273
HCT (%)	38.77 ± 5.01	39.32 ± 3.29	0.485
Platelets (x10^3^/μL)	233.89 ± 62.81	221.05 ± 59.62	0.286
NEU (%)	46.73 ± 14.44	47.89 ± 10.64	0.649
LYM (%)	44.33 ± 14.43	43.80 ± 9.50	0.816

WBC-White blood cells; RBC-Red blood cells; HGB-Haemoglobin; HCT-Haematocrit; NEU-Neutrophils; LYM-Lymphocytes (* *p*-value <0.05 is considered significant); SD, standard deviation

#### Correlation between SOD and haematological parameters

In Table 4, association between SOD activity and haematological changes are compared. Higher neutrophil activity seemed to weakly correlate with increased SOD activity (R = 0.249; R^2^ = 6.2 %; p < 0.049). None of the other measured haematological indices correlated with SOD activity values. Similarly, there did not appear to be correlation between FBG and SOD activity.

**Table 4:** Correlation between SOD Activity Levels and haematological indices

Pearson’s Correlations	R	*p* value
FBG and SOD Levels	−0.044	0.727
WBC and SOD Levels	0.047	0.714
RBC and SOD Levels	0.113	0.375
HGB and SOD Levels	0.028	0.824
HCT and SOD Levels	0.002	0.99
MCV and SOD Levels	−0.092	0.469
MCH and SOD Levels	−0.078	0.542
MCHC and SOD Levels	0.004	0.975
PLT and SOD Levels	0.235	0.061
NEU(%) and SOD Levels	0.249*	0.049*
LYM(%) and SOD Levels	−0.208	0.099

*Correlation is significant at p value < 0.05 (2-tailed)

WBC-White blood cells; RBC-Red blood cells; HGB-Haemoglobin; HCT-Haematocrit; NEU-Neutrophils; LYM-Lymphocytes; FBG- Fasting blood glucose; SOD- Superoxide dismutase; PLT-platelet count; NEU-neutrophil count; LYM- lymphocyte count; MCV- Mean corpuscular volume; MCH- Mean corpuscular haemoglobin; MCHC- Mean corpuscular haemoglobin concentration

## Discussion

Oxidative stress is increasingly being reported as a widely accepted participant in the progression of diabetes and its complications. In this study, the mean fasting plasma glucose level of patients with diabetes was 8.34 mmol/l (Table 2) — significantly higher than the recommended target FPG indicative of good glycaemic control (3.9 – 7.2 mmol/l) — and confirming that the cases were more likely to be exposed to persistent hyperglycaemia [10, 11]. Persistent hyperglycaemia in diabetes is often accompanied by increased production of free radicals or impaired antioxidant defenses [7, 12–14]. In the present study, the SOD activity tended to be lower in the diabetes group (p > 0.05 however) compared to controls but the trend did not achieve statistical significance. Majority of the diabetic cases were on metformin (either as monotherapy or in combination with other medications such as insulin) and most likely may have benefited from their antioxidant properties. Metformin, a drug taken to control diabetes has been reported to possess antioxidant properties by inhibiting generation of reactive oxidative species in diabetes and thereby reducing oxidative stress [15]. A reduction in ROS levels in human leukocytes due to metformin administration by direct scavenging of the free radicals has been reported by Bonnefont-Rousselot *et al*. [16]. Other hypoglycaemic agents such as insulin have also been shown to have similar impact on oxidative stress [17].

Changes in haematological indices, except for the higher WBC trend in the diabetes group, were similar in both study cohorts. It is noteworthy that the high white cell count in the diabetes group is in keeping with the increased oxidative stress triggered by the high levels of hyperglycaemia observed in this cohort. Polymorphonuclear and mononuclear leukocytes can be activated by advanced glycation end-products and cytokines in a state of hyperglycaemia [18].

Our study reports of SOD activity that significantly albeit weakly correlated with neutrophil count. The observation agree well with findings by Kotani and Sakane [19] who worked on diacron reactive oxygen metabolites (d-ROMs) as clinical markers for evaluation of total oxidative burden. Their work showed positive correlation between neutrophil counts and d-ROMs levels in asymptomatic female subjects. Neutrophils secrete a variety of inflammatory cytokines such as interleukin-6 which trigger an increase in oxidative stress [20, 21]. Oxidative stress also activates the inflammatory pathway with leukotriene B4 which attract neutrophils to inflammatory sites further generating oxidative stress [19, 22].

There are some potential limitations of this study that should be discussed briefly. First, the limited sample size. This could partly explain our findings of similar SOD activity values in the diabetic and control cohorts. A more large-scale survey is likely to be more accurate with little bias for unequal drug preferences. Another noteworthy limitation to our study is its cross-sectional design involving single measurement of haematological indices which may not demonstrate a temporal relationship with oxidative stress. Additionally intercurrent infections which were not actively sought for by our study cannot be completely excluded and might have potentially exerted confounding effects on the haematological indices.

## Conclusion

In conclusion, we report similar SOD activity and for that matter similar oxidative stress levels in diabetic and non-diabetic adults. Oxidative stress as measured by SOD activity did not appear to correlate with FBG and several other haematological parameters. Further study would be required to investigate the relationship between these haematological indices and diabetic micro and macrovascular complications in our population.

## Abbreviations

DM: Diabetes Mellitus
ROS: reactive oxygen species
NDMRC: National Diabetes Management and Research Centre
SOD: Superoxide dismutase
GPx: glutathione peroxidase
CAT: catalase
GSH: reduced gluthathione
DNA: Deoxyribonucleic acid
OGTT: Oral-Glucose-Tolerance-Test
HBA1C: glycated haemoglobin A1c
FPG: fasting plasma glucose
FBC: full blood count
Cu/ZnSOD: Copper/Zinc Superoxide dismutase
SPSS: Statistical Package for Social Sciences
O2•-: superoxide
O2: oxygen
RBC: Red blood cells
d-ROMs: diacron reactive oxygen metabolites
